# Supplementary material for: Thyroid Dysfunction under Amiodarone in Patients with and without Congenital Heart Disease: Results of a Nationwide Analysis
Source: J Clin Med. 2022 Apr 5;11(7):2027. doi: 10.3390/jcm11072027 (PMC8999848; doi:10.3390/jcm11072027)
Supplement: Supplementary file 1 [file jcm-11-02027-s001.zip › jcm-1591312-supplementary.pdf]

**Supplementary Materials:**

**Table S1:** ICD-10-GM Codes/ OPS diagnosis and procedure codes/ATC codes relevant for analysis.

| | |
|---|---|
| **Antiarrhythmic drugs** | |
| Class Ia | ATC: C01BA |
| Class Ib | ATC: C01BB |
| Class Ic | ATC: C01BC |
| Class III excluding amiodarone | ATC: C01BCD excluding C01BD01 |
| Amiodarone | ATC: C01BD01 |
| **Arrhythmia** | |
| Atrial re-entrant tachycardia | ICD-10-GM: I47.1 |
| Atrial fibrillation | ICD-10-GM: I48.0 - I48.2 |
| Atrial flutter | ICD-10-GM: I48.3, I48.4 |
| Ventricular re-entrant tachycardia | ICD-10-GM: I47.0 |
| Ventricular tachycardia (other) | ICD-10-GM: I47.2 |
| Ventricular extrasystole | ICD-10-GM: I49.3 |
| Ventricular flutter/ fibrillation | ICD-10-GM: I49.0 |
| Cardiac arrest (not specified further) | ICD-10-GM: I46.1, I46.1, I46.9 |
| **Baseline characteristics** | |
| Left heart failure | ICD-10-GM: I50.1 |
| Right heart failure | ICD-10-GM: I50.0 |
| Implantation of a pacemaker | OPS: 5-377.0- 5-377.4 |
| Implantation of an implantable cardioverter defibrillator | OPS: 5-377.5, 5-377.7 |
| Obesity | ICD-10-GM: E66 |
| Smoking | ICD-10-GM: F17 |
| Alcohol abuse | ICD-10-GM: F10 |
| Chronic kidney disease | ICD-10-GM: N18.4, N18.5 |
| Liver dysfunction | ICD-10-GM: K72.1, K72.7 |
| **Heart failure drug therapy** | |
| ACE-Inhibitors/ Angiotensin II receptor blockers | ATC: C09 |
| Diuretics | ATC: C03 |
| Betablockers excluding sotalol | ATC: C07A excluding C07AA07 |
| Cardiac glycosides | ATC: C01AA |
| Calcium channel blockers | ATC: C08 |
| **Thyroid dysfunction** | |
| Hyperthyroidism | ICD-10-GM: E05 |
| Hypothyroidism | ICD-10-GM: E03 |
| Thyroiditis | ICD-10-GM: E06 |
| Thyroid radiotherapy | OPS: 8- 53 |
| Thyroid surgery | OPS: 5- 06 |
| **Drug therapy/catheter ablation** | |
| Catheter ablation | OPS: 1-26 |
| Levothyroxine | ATC: H03AA01 |
| Thiamazole | ATC: H03BB02 |
| Propylthiouracil | ATC: H03BA02 |
| Sodiumperchlorate | ATC: H03BC02 |

**Table S2:** ICD-10 GM-Codes used for identification and grouping of patients with congenital heart disease (CHD).

| | |
|---|---|
| **Simple CHD** | |
| Isolated ventricular septal defect | Q21.0 |
| Persistent arterial duct | Q25.0 |
| Isolated congenital valve disease | Q23.0, Q23.1, Q22.4, Q22.8, Q22.9, Q23.2, Q23.3, Q22.1, Q22.2, Q22.3 |
| Other congenital malformation of the great arteries | Q25.8, Q25.9 |
| **Moderate complexity CHD** | |
| Tetralogy of Fallot | Q21.3, Q21.80, (Q22.0 and Q21.0) |
| Ebstein's anomaly | Q22.5 |
| Aortic isthmus stenosis, interrupted aortic arch | Q25.1, Q25.2 |
| Atrioventricular septal defect | Q21.2 |
| Partial anomalous pulmonary venous connection | Q26.3, Q26.4 |
| **Severely complex CHD** | |
| Univentricular heart | Q20.1, Q20.2, Q20.4, Q22.6, Q23.4, (Q22.0 without Q21.0) |
| Eisenmenger's syndrome | I27.8 and at least one further Q-Code with the exception of Q21.1<br>or Q21.88 and at least one further Q-Code with the exception of Q21.1 |
| Transposition of the great arteries (TGA) | Q20.3, Q20.5 |
| Other complex heart malformation, e.g. total anomalous pulmonary venous connection, common arterial trunk | Q20.0, Q26.2 |

**Figure S1:** Study design.

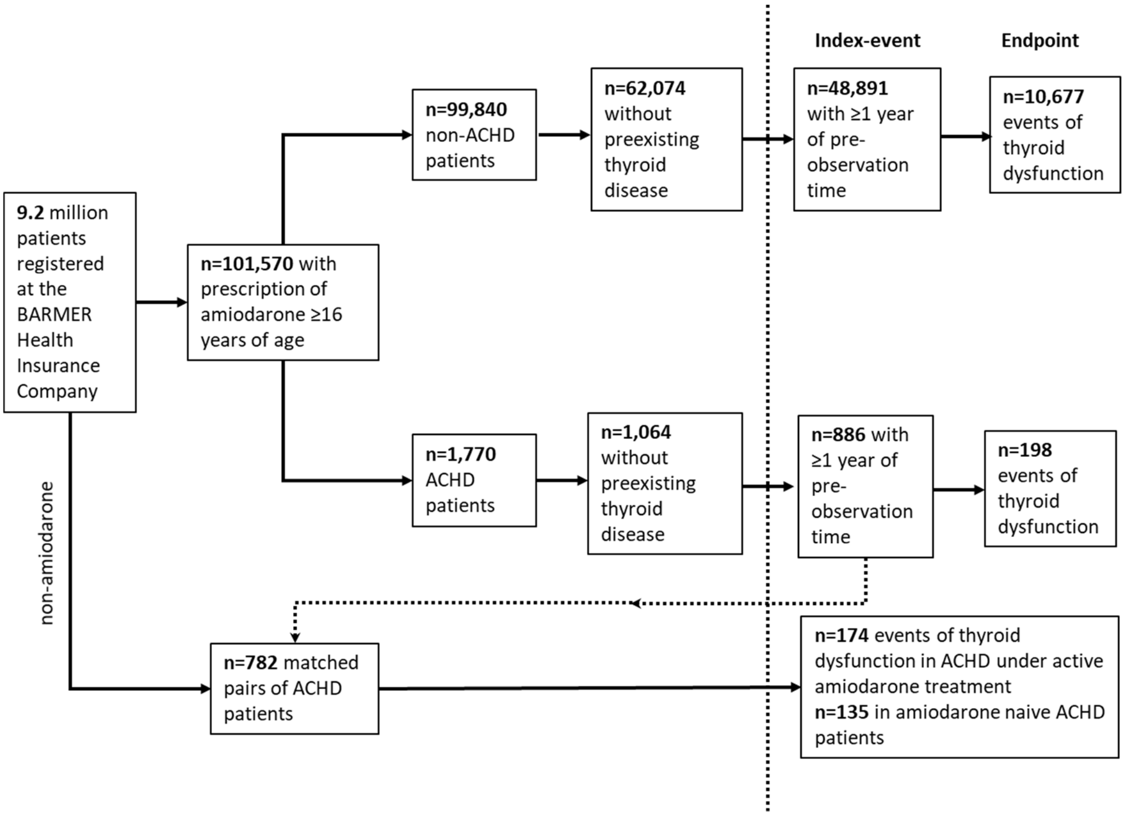

**Table S3:** Risk factors for occurrence of **hyperthyroidism** after amiodarone intake in **all patients;** results of the multivariable Cox regression analysis.
*active amiodarone intake defined as prescription of amiodarone within 90 days before and after analyzation time. To avoid the problem of non-proportional hazards, a stepwise analysis showing yearly intervals was used, thus allowing the corresponding coefficient to be time-varying.

| **Variable** | **Hazard ratio (95% CI)** | ***p*-value** |
|---|---|---|
| Active amiodarone intake (year 1)* | 1.45 (1.23-1.71) | **<0.001** |
| Active amiodarone intake (year 2)* | 1.40 (1.23-1.59) | **<0.001** |
| Active amiodarone intake (year 3)* | 2.70 (2.37-3.07) | **<0.001** |
| Active amiodarone intake (year ≥4)* | 3.33 (2.99-3.70) | **<0.001** |
| Age /10 years | 0.89 (0.86-0.91) | **<0.001** |
| Female gender | 1.13 (1.06-1.19) | **<0.001** |
| Congenital heart disease | 0.95 (0.78-1.15) | 0.60 |
| Pacemaker therapy | 1.06 (0.96-1.17) | 0.27 |
| Implantable cardioverter defibrillator | 1.15 (1.05-1.26) | **0.003** |
| Chronic kidney disease | 0.90 (0.75-1.09) | 0.28 |
| Liver dysfunction | 0.83 (0.26-2.61) | 0.75 |
| Alcohol abuse | 0.96 (0.84-1.08) | 0.48 |
| Smoking | 1.13 (1.04-1.22) | **0.004** |
| Obesity | 0.88 (0.83-0.93) | **<0.001** |

**Table S4:** Risk factors for occurrence of **hyperthyroidism** after amiodarone intake in adults with **congenital heart disease only** (CHD); results of the multivariable Cox regression analysis.
*active amiodarone intake defined as prescription of amiodarone within 90 days before and after analyzation time. To avoid the problem of non-proportional hazards, a stepwise analysis showing yearly intervals was used, thus allowing the corresponding coefficient to be time-varying.

| **Variable** | **Hazard ratio (95% CI)** | ***p*-value** |
|---|---|---|
| Active amiodarone intake (year 1)* | 1.01 (0.33-3.10) | 0.98 |
| Active amiodarone intake (year 2)* | 2.09 (1.00-4.40) | 0.05 |
| Active amiodarone intake (year 3)* | 4.21 (1.61-10.99) | **0.003** |
| Active amiodarone intake (year ≥4)* | 4.12 (2.02-8.40) | **<0.001** |
| Age /10 years | 0.93 (0.82-1.07) | 0.32 |
| Female gender | 1.36 (0.90-2.05) | 0.15 |
| Medium complexity CHD vs. simple | 1.06 (0.67-1.68) | 0.81 |
| High complexity CHD vs. simple | 1.33 (0.74-2.39) | 0.33 |
| Pacemaker therapy | 1.39 (0.77-2.49) | 0.27 |
| Implantable cardioverter defibrillator | 1.32 (0.72-2.41) | 0.37 |
| Alcohol abuse | 0.28 (0.04-2.16) | 0.22 |
| Smoking | 0.93 (0.48-1.79) | 0.82 |
| Obesity | 0.79 (0.49-1.27) | 0.33 |

**Table S5:** Risk factors for occurrence of hypothyroidism after amiodarone intake in all patients; results of the multivariable Cox regression analysis.
*active amiodarone intake defined as prescription of amiodarone within 90 days before and after analyzation time. To avoid the problem of non-proportional hazards, a stepwise analysis showing yearly intervals was used, thus allowing the corresponding coefficient to be time-varying.

| **Variable** | **Hazard ratio (95% CI)** | ***p*-value** |
|---|---|---|
| Active amiodarone intake (year 1)* | 2.44 (2.05-2.90) | **<0.001** |
| Active amiodarone intake (year 2)* | 2.31 (2.02-2.64) | **<0.001** |
| Active amiodarone intake (year 3)* | 2.09 (1.82-2.40) | **<0.001** |
| Active amiodarone intake (year ≥4)* | 2.87 (2.66-3.10) | **<0.001** |
| Age /10 years | 1.04 (1.01-1.07) | **0.005** |
| Female gender | 1.63 (1.56-1.72) | **<0.001** |
| Congenital heart disease | 1.08 (0.91-1.28) | 0.36 |
| Pacemaker therapy | 1.12 (1.03-1.22) | **0.005** |
| Implantable cardioverter defibrillator | 1.38 (1.27-1.49) | **<0.001** |
| Chronic kidney disease | 1.89 (1.68-2.12) | **<0.001** |
| Liver dysfunction | 0.57 (0.19-1.75) | 0.33 |
| Alcohol abuse | 1.45 (1.31-1.60) | **<0.001** |
| Smoking | 1.11 (1.03-1.20) | **0.005** |
| Obesity | 1.11 (1.06-1.16) | **<0.001** |

**Table S6:** Risk factors for occurrence of **hypothyroidism** after amiodarone intake in **adults with congenital heart disease only** (CHD); results of the multivariable Cox regression analysis.

*active amiodarone intake defined as prescription of amiodarone within 90 days before and after analyzation time. To avoid the problem of non-proportional hazards, a stepwise analysis showing yearly intervals was used, thus allowing the corresponding coefficient to be time-varying.

| **Variable** | **Hazard ratio (95% CI)** | ***p*-value** |
|---|---|---|
| Active amiodarone intake (year 1)* | 3.37 (0.97-11.74) | 0.06 |
| Active amiodarone intake (year 2)* | 2.36 (1.12-5.00) | **0.02** |
| Active amiodarone intake (year 3)* | 2.47 (0.85-7.21) | 0.10 |
| Active amiodarone intake (year ≥4)* | 2.39 (1.40-4.11) | **0.002** |
| Age/10 years | 0.87 (0.77–0.99) | **0.03** |
| Female gender | 1.79 (1.27-2.52) | **0.001** |
| Medium complexity CHD vs. simple | 1.27 (0.86-1.89) | 0.23 |
| High complexity CHD vs. simple | 1.73 (1.11-2.70) | **0.02** |
| Pacemaker therapy | 1.27 (0.74-2.18) | 0.39 |
| Implantable cardioverter defibrillator | 1.29 (0.71-2.33) | 0.41 |
| Chronic kidney disease | 2.97 (1.16-7.59) | **0.02** |
| Alcohol abuse | 1.31 (0.54-3.18) | 0.55 |
| Smoking | 1.41 (0.87-2.31) | 0.17 |
| Obesity | 1.06 (0.73-1.54) | 0.77 |

**Table S7:** Use of catheter ablation and antiarrhythmic drug treatment around the time of diagnosis of thyroid dysfunction in adults with congenital heart disease (ACHD) versus non-ACHD.

| | **Non-ACHD, n=48,891** | | **ACHD, n=886** | |
|---|---|---|---|---|
| | **6 months before endpoint** | **6 months after endpoint** | **6 months before endpoint** | **6 months after endpoint** |
| **Combined endpoint thyroid dysfunction, n (%)** | 10,677 (21.8) | | 198 (22.3) | |
| Catheter ablation, n (%) | 858 (8.0) | 1,352 (12.7) | 23 (11.6) | 31 (15.7) |
| Intake of antiarrhythmic drugs | | | | |
| Class Ia, n (%) | 5 (0.0) | 4 (0.0) | 0 (0.0) | 0 (0.0) |
| Class Ib, n (%) | 9 (0.1) | 6 0.1) | 0 (0.0) | 0 (0.0) |
| Class Ic, n (%) | 199 (1.9) | 221 (2.1) | 7 (3.5) | 8 (4.0) |
| Class III, n (%) | 7,904(74.0) | 5,171 (48.4) | 129 (65.2) | 80 (40.4) |
| Amiodarone only, n (%) | 7,842 (73.4) | 5,022 (47.0) | 128 (64.6) | 75 (37.9) |
| **Hyperthyroidism,** n (%) | 5,094 (10.4) | | 103 (11.6) | |
| Catheter ablation, n (%) | 425 (8.3) | 714 (14.0) | 13 (12.6) | 17 (16.5) |
| Intake of antiarrhythmic drugs | | | | |
| Class Ia, n (%) | 3 (0.1) | 3 (0.1) | 0 (0.0) | 0 (0.0) |
| Class Ib, n (%) | 5 (0.1) | 5 (0.1) | 0 (0.0) | 0 (0.0) |
| Class Ic, n (%) | 118 (2.3) | 149 (2.9) | 3 (2.9) | 6 (5.8) |
| Class III, n (%) | 3,613 (70.9) | 1,990 (39.1) | 64 (62.1) | 32 (31.1) |
| Amiodarone only, n (%) | 3,574 (70.2) | 1,882 (36.9) | 64 (62.1) | 29 (28.2) |
| **Hypothyroidism,** n (%) | 7,079 (14.5) | | 138 (15.6) | |
| Catheter ablation, n (%) | 587 (8.3) | 863 (12.2) | 16 (11.6) | 22 (15.9) |
| Intake of antiarrhythmic drugs | | | | |
| Class Ia, n (%) | 2 (0.0) | 1 (0.0) | 0 (0.0) | 0 (0.0) |
| Class Ib, n (%) | 7 (0.1) | 4 (0.1) | 0 (0.0) | 0 (0.0) |
| Class Ic, n (%) | 117 (1.7) | 109 (1.5) | 3 (2.2) | 1 (0.7) |
| Class III, n (%) | 4,978 (70.3) | 3,628 (51.3) | 82 (59.4) | 61 (44.2) |
| Amiodarone only, n (%) | 4,925 (69.6) | 3,554 (50.2) | 81 (58.7) | 59 (42.8) |
